# Endoscopic Submucosal Dissection, Endoscopic Mucosal Resection, and Transanal Minimally Invasive Surgery for the Management of Rectal and Anorectal Lesions: A Narrative Review

**DOI:** 10.3390/jcm12144777

**Published:** 2023-07-19

**Authors:** Pedro Moreira, Pedro Marílio Cardoso, Guilherme Macedo, João Santos-Antunes

**Affiliations:** 1 Faculty of Medicine, University of Porto, 4200-450 Porto, Portugal; pedrom.moreira98@gmail.com (P.M.); pedromarilio@gmail.com (P.M.C.); guilhermemacedo59@gmail.com (G.M.); 2 Gastroenterology Department, Centro Hospitalar São João, 4200-319 Porto, Portugal; 3 i3S-Instituto de Investigação e Inovação em Saúde, University of Porto, 4099-030 Porto, Portugal; 4 IPATIMUP-Institute of Molecular Pathology and Immunology, University of Porto, 4200-450 Porto, Portugal

**Keywords:** endoscopic submucosal dissection, endoscopic mucosal resection, transanal minimally invasive surgery, rectal adenoma, rectal cancer, rectal polyps, anorectal junction, laterally spreading tumors

## Abstract

Endoscopic submucosal dissection (ESD), endoscopic mucosal resection (EMR), and transanal minimally invasive surgery (TAMIS) are modern techniques that now play a crucial role in the treatment of colorectal lesions. ESD is a minimally invasive endoscopic procedure that allows for the resection of lesions of any size in a single piece, with clear advantages regarding oncological outcomes and recurrences. However, it is a complex technique, requiring high endoscopic skills, expertise, and specialized training, with higher rates of adverse events expected compared with EMR. EMR is another endoscopic technique used to remove superficial gastrointestinal tumors, particularly those that are limited to the mucosal layer. It is a faster and more accessible procedure, with fewer adverse events, although it only allows for an en-bloc resection of lesions measuring 15–20 mm. TAMIS is a minimally invasive surgical technique used to remove rectal tumors, involving the insertion of a single-port device through the anus, allowing for a better visualization and removal of the tumor with minimal disruption. This article reviews the current applications and evidence regarding these techniques, in search for the most adequate treatment for the removal of lesions in the rectum and anorectal junction, as these locations possess distinct characteristics that demand a more specific approach.

## 1. Introduction

Gastrointestinal neoplasia is a common cause of morbidity and mortality worldwide.

Colorectal cancer (CRC) is the third most deadly and fourth most commonly diagnosed cancer in the world, with an increased incidence worldwide, especially in developing countries that are adopting the Western lifestyle. Obesity, a sedentary lifestyle, red meat consumption, alcohol, and tobacco are prevalent factors that increase the risk of this neoplastic condition [1].

Despite advances in screening and treatment, CRC remains a leading cause of cancer-related deaths. To reduce the mortality and morbidity associated with CRC, a strategy for the early detection of pre-malignant or early malignant lesions has been developed. If detected early, these lesions can be removed using less invasive techniques instead of resorting to the conventional colectomy, lower anterior rectal resection, or abdomino-perineal amputation. These less invasive techniques are especially useful in cases of rectal cancer, where the lesions are more prone to having advanced or aggressive features [2,3], and the technical difficulty of the anorectal junction (ARJ) makes surgical intervention more challenging [4]. Among these less invasive techniques are endoscopic submucosal dissection (ESD), endoscopic mucosal resection (EMR), and transanal minimally invasive surgery (TAMIS).

For this narrative review, a literature search using electronic databases (MEDLINE/PubMed, EMBASE, and Cochrane Library) was performed. Articles about EMR, ESD, and TAMIS were collected, and outcomes were registered in order to discuss their effectiveness in treating rectal and anorectal conditions, as well as potential future directions for these techniques. Selected literature types included research articles, systematic reviews, narrative reviews, and letters. Studies not written in English or Portuguese were excluded. The terms “endoscopic submucosal dissection”, “endoscopic mucosal resection”, “transanal minimally invasive cancer”, “rectal cancer”, “anorectal cancer”, and “adenoma detection” were mainly, but not exclusively, used as search terms (Table 1).

## 2. Early Colorectal Cancer

CRC usually progresses through several stages, starting from normal epithelium to a pre-malignant lesion known as adenoma, and finally to a malignant lesion or adenocarcinoma, which can invade surrounding tissues and spread systemically through metastasis. It is estimated that about 5% of adenomas progress to cancer, requiring the usage of screening strategies to identify premalignant lesions and remove them [5,6].

The Japanese [7], European [8], and American [9] guidelines recommend that large sessile colorectal polyps and laterally spreading tumors (LSTs) can be removed by piecemeal endoscopic mucosal resection (p-EMR) if there are no signs of submucosal invasion upon endoscopic assessment. The most suspicious area should be removed via en-bloc resection, while the remaining lesion is deliberately removed in a piecemeal fashion. However, though EMR is a fast and safe method for removing superficial non-pedunculated colorectal lesions sized above 10–15 mm [3,10], it may hinder accurate histological assessment and has higher recurrence rates than en-bloc resection [7,9,11,12], resulting in a higher frequency of post-procedural surgery [3,4].

In recent years, ESD has revolutionized the field of gastroenterology by offering minimally invasive approaches for the treatment of gastrointestinal neoplasia, with high rates of en-bloc resection and a lower recurrence rate when compared to EMR [12,13,14]. This technique first appeared in Japan for the treatment of gastric lesions but is currently used for the removal of lesions in other organs in an en-bloc fashion, regardless of the lesion’s size, leading to a minimized recurrence risk [15]. However, due to the colon tortuosity, poor maneuverability of the endoscope when compared to other organs, and the results of large EMR series [16,17], the use of ESD in the colorectum is still controversial. It is more technically demanding and time-consuming, and there is a steep learning curve and a higher rate of adverse events, particularly in the West [13,18]. Moreover, despite the continuity of the colon and rectum, the lesions in these areas should probably not be approached by the same algorithm, considering the increased rate of submucosal invasive malignancy in the rectum [2,3].

Besides the endoscopic techniques of EMR and ESD, TAMIS is a minimally invasive surgical approach that is also accomplishing satisfactory results. It provides a means of accessing the proximal and mid-rectum for the removal of benign and early-stage malignant rectal lesions. It can also be employed for the non-radical surgical treatment of more advanced lesions in patients for whom radical surgery is not suitable. It has a shallower learning curve, a quicker device setup time, and more flexibility and versatility in instrument application compared to transanal endoscopic microsurgery (TEM), while the operation time, conversion, reoperation, and complication rates between TAMIS and TEM are comparable [19,20]. Therefore, a comprehensive analysis of this technique, together with those endoscopic techniques, is warranted, in order to allow for an evidence-based selection of the best minimally invasive treatment for rectal lesions.

## 3. An Evaluation of Rectal Lesion Candidates for Endoscopic Resection

Primarily, it is necessary to assess which lesions can be managed by endoscopy techniques. Colorectal lesion morphology characterization can help predict the risk of submucosal invasive carcinoma (SMIC), and it helps in selecting the most appropriate endoscopic treatment [8,9,21,22]. Several classification systems that analyze morphology, surfaces, and microvasculature have been developed for this purpose and are known to help predict malignancy and deep submucosal invasion depth. Some of the most studied are the Paris and LST classifications for morphology [23,24], pit patterns (PPs) according to the Kudo classification for surface evaluations [25], and NICE and JNET for vascular patterns [23,24].

**Figure 1:**
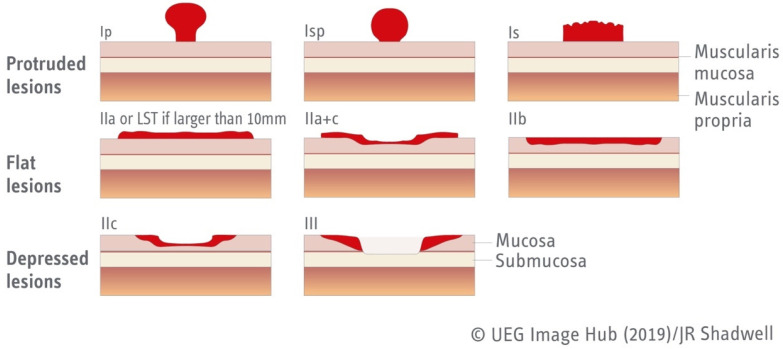
Paris classification of type 0 lesions [23]. Figure adapted from UEG Image Hub.

In terms of morphology, the Paris classification categorizes superficial gastrointestinal neoplastic lesions in five categories (Type 0–5) based on their morphology and depth of invasion. Only Type 0 lesions are within the scope of this article since Type 1–5 correspond to advanced malignancy. Type 0 lesions are then subclassified based on their morphology, being further categorized as 0-Is, 0-Ip, and 0-Isp (protruded lesions), 0-IIa, 0-IIb, and 0-IIc for non-protuded/non-excavated lesions, or Type III for excavated lesions (Figure 1) [23].

**Figure 2:**
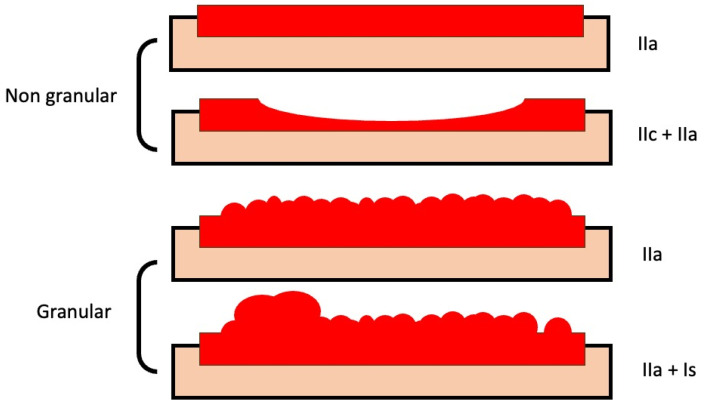
Subtypes of laterally spreading tumors. Models of granular and non-granular LSTs of neoplastic lesions (with flat-elevated and pseudo-depressed subtypes in non-granular lesions, and homogeneous and mixed-nodular subtypes in granular lesions), with corresponding classifications according to the categories of the Paris classification.

Nonpolypoid lesions with a lateral growth larger than 10 mm are called LSTs [26] and are classified into two main types according to their morphology: granular (LST-G) and non-granular (LST-NG). Each type has two subtypes (Figure 2): granular LSTs can be "homogenous" (LST-GH) or "mixed nodular" (LST-GMN) lesions, while non-granular LSTs can be "flat elevated" (FE) or "pseudodepressed" (LST-NGPD) lesions. LST-GHs are the lesions with a lower probability of harboring malignancy (an SMIC risk of 0.5%), so most of them can be effectively treated by p-EMR. However, LST-NGPDs have the highest risk of malignancy (as high as 32%), so an en-bloc resection must be pursued to ensure an accurate pathological evaluation and a higher probability of cure; this is accomplished mainly by ESD. Similarly, in LST-GMNs, the presence of a large nodule has been linked with an increased occurrence of SM invasion and must be resected in one fragment, while the remaining flat component can be removed in a piecemeal fashion in the absence of other suspicious areas. These lesions comprise up to one-quarter of LSTs and are considered as having an intermediate risk of covert SMIC; submucosal invasion occur mostly under large nodules (84%) and depressed areas (16%) [27].

Besides morphology, an assessment of surface and vascular patterns should be performed using a high-definition endoscope, and optical magnification is sometimes required [21,23,24]. In fact, guidelines recommend nowadays the use of high-resolution colonoscopy with chromoendoscopy and optical magnification to establish the presence of SMIC and to evaluate the possibility of resection [23,24,25]. Chromoendoscopy with indigo carmine or crystal violet is the preferred method for assessing the surface of the lesions (pit patterns) and thus predicting the risk of submucosal invasion in Japan (Kudo classification). Through real-time imaging modifications such as narrow-band imaging (NBI), flexible spectral imaging color enhancement (FICE), or i-Scan, virtual chromoendoscopy also enables an accurate evaluation of pit patterns as well as vascular patterns [9,21,23,25]. 

Optical magnification endoscopes are used to identify the mucosal surface PPs according to the Kudo classification system [7,28]. The Kudo PP classification system categorizes the surface pattern of colorectal polyps into four types based on the morphology of pits or crypts on the surface of polyps [28]. These patterns are classified as follows: Type I: round or oval pits that are regular in size and distribution; Type II: round or oval pits that are larger and less regular than Type I; Type III: tubular or branched pits that are irregular in size and distribution; Type IV: villous or cerebriform appearance with no pits. Type V can be further classified into two subgroups: Type Vi (I: irregular) and Type Vn (N: non-structured). Type Vi indicates the presence of histological structural atypia outgrowth of cancerous glands, while Type Vn lacks a superficial microstructure and represents the exposure of a desmoplastic reaction from deeply invasive submucosal components to the surface. This classification system is useful in predicting the histology of colorectal polyps and can aid in determining the most appropriate resection technique and predicting the likelihood of an incomplete resection. In contrast, Western areas still tend to rely on the lifting sign and NBI due to the limitations of magnification availability, higher costs, and longer procedural times. As a result, the evaluation of lesion infiltration risk may not be as precise in Western areas as it is in Japan [8,21,23,25].

The early detection of CRC has been significantly enhanced by the use of advanced technologies, which enable the characterization of the microscopic features of the dimples or furrows that divide the mucosal cells, as well as the microvasculature around the pits. These features change in accordance with the different stages of dysplasia and neoplastic transformation [9,21,23,25]. With the help of NBI, the sensitivity and specificity for diagnosing T1 CRC with SMIC have been reported as 77% and 98%, respectively [29].

The NICE classification system [30], which comes from the employment of NBI, enables the differentiation between hyperplastic polyps (Type 1), adenomas or adenocarcinomas with superficial submucosal invasion (Type 2), and cancers with deep submucosal invasion (Type 3) based on various features such as color, vessels, and surface patterns [30,31,32]. 

The combination of these classification systems can help in deciding the best approach. Hence, lesions that display glandular distortion with preserved vascular structures (Kudo Vi and NICE Type 2) are probably superficial SMIC and are appropriate for endoscopic en-bloc resection. On the other hand, a severely distorted polyp or an absence or irregularity of submucosal vessels (Kudo Vn or NICE Type 3) indicates a high likelihood of deep SMIC, and a surgical approach is warranted. 

In a study that evaluated the accuracy of the NBI identification of deep invasion, the results showed a sensitivity of 59% and a specificity of 96% for predicting deep SMIC, with a positive predictive value (PPV) of 58% and a negative predictive value (NPV) of 98% [32]. In a randomized controlled trial that also evaluated the NICE classification system demonstrated a high level of accuracy in differentiating neoplastic from non-neoplastic polyps, with a specificity of 99%, a sensitivity of 58%, a PPV of 95%, and a NPV of 88% [31].

The JNET classification [33] is a diagnostic system for hyperplastic/sessile serrated polyps (Type 1), neoplasia with low intramucosal neoplasia (Type 2A), high-grade intramucosal neoplasia/shallow SMIC (Type 2B), and cancer with deep SMIC (Type 3) [34,35,36], based on vascular and superficial patterns subdividing Group 2 of the NICE classification system. Both non-expert and expert endoscopists had similar specificity, NPV, and accuracy (>90%) for Type 1, 2B, and 3, with a sensitivity and PPV above 90% for Type 2A. Type 2B exhibited a sensitivity of only 42% [37].

Colorectal polyps with ulceration, excavation, defined deep depression, Paris IIc and IIa+c, mucosal friability, convergent plicae, and Kudo Type V PPs were found to likely correspond to SMIC, putting them at high risk for lymphovascular invasion and lymph node metastasis. Rectal tumors with suspected SMIC and possible lymph node involvement based on endoscopic features can undergo staging using endoscopic ultrasound and magnetic resonance imaging [38,39].

When considering the endoscopic removal of lesions, the presence of non-lifting signs is a significant factor that warrants attention. Non-lifting is often an indirect indication of deep submucosal invasion and may represent contraindication to endoscopic removal [40,41]. Other factors that may contribute to non-lifting signs include the presence of fibrotic tissue observed in some lesions, mainly in LST-NGPDs, or previous manipulations with submucosal injection or biopsies [41]. Furthermore, it is important to notice that SMIC involving the upper and middle level of the submucosa (SM1 and SM2, respectively) is not strongly associated with non-lifting signs, as the underlying undamaged submucosa can still expand [24,40,41,42]. In contrast, deep SMIC involving the lower level of the submucosa (SM3) is more likely to present with non-lifting signs. Therefore, in cases where deep SMIC is suspected or confirmed, surgery is indicated.

### 3.1. Rectal and Anorectal Junction Lesions: How They Differ in Terms of Malignancy Risk and Treatment Approach

#### 3.1.1. Rectal Lesions

The rectum and colon are in continuity; thus, generally, the lesions in these areas are subjected to the same endoscopic treatment algorithm. However, this might not be the correct approach since, in the rectum and ARJ, the rate of malignancy incidence is higher when compared to colonic lesions [2,3]. The rate of covert SMIC (i.e., no clear signs of invasion) among LSTs in this location is higher than in the colon. In the article written by D´amico et al. [3], the incidence of submucosal invasive cancer in LST-GMNs is approximately 75% higher in the rectum when compared to the colon. As there are no clear signs of invasion, it is challenging to determine which LST-GMNs require en-bloc resection. As the study showed a 22% risk of covert SMIC for >4 cm rectal LST-GMNs, such a finding justifies the need for a more effective treatment, in an en-bloc fashion. Besides the location of the lesion, size is another important risk factor, with larger lesions indicating a higher malignancy rate. It has been shown that rectal large non-pedunculated colorectal polyps (LNPCPs) tend to be larger when compared to colonic polyps and that there is also an increased risk of neoplastic lesion transformation. Rectal LNPCPs were also shown to be more likely to have tubulovillous histopathology and to show signs of submucosal invasion [2]. Therefore, despite the similarities between the colon, rectum, and anorectal junction, there are significant differences regarding malignancy risk, with obvious implications for endoscopic treatment, which demands a distinct approach [43,44].

#### 3.1.2. Anorectal Junction Lesions

The anorectal junction is located in the distal part of the gastrointestinal tract, positioned within a distance of less than 2 cm from the pectineal line, where the transitional zone is located, demonstrating a combination of columnar, cuboidal, transitional, and squamous epithelial cells [45]. Endoscopic resection at the ARJ is technically challenging due to the presence of distinctive anatomic and physiologic characteristics [14]. There is a unique innervation due to the anoderm proximity and lymphovascular supply due to the rectal venous plexus. This requires specific measures to limit pain and bleeding from resecting over the dentate line and hemorrhoidal vessels. There is also a theoretical risk of systemic bacteremia because of direct drainage into the systemic circulation [4,14]. Surgery is also not the best procedure in this area due to the risk of damaging the anal function and other complications such as anal stenosis, fecal incontinence, and chronic pelvic pain, which can significantly influence the patient’s quality of life [46].

## 4. Minimally Invasive Techniques

### 4.1. Endoscopic Mucosal Resection

**Figure 3:**
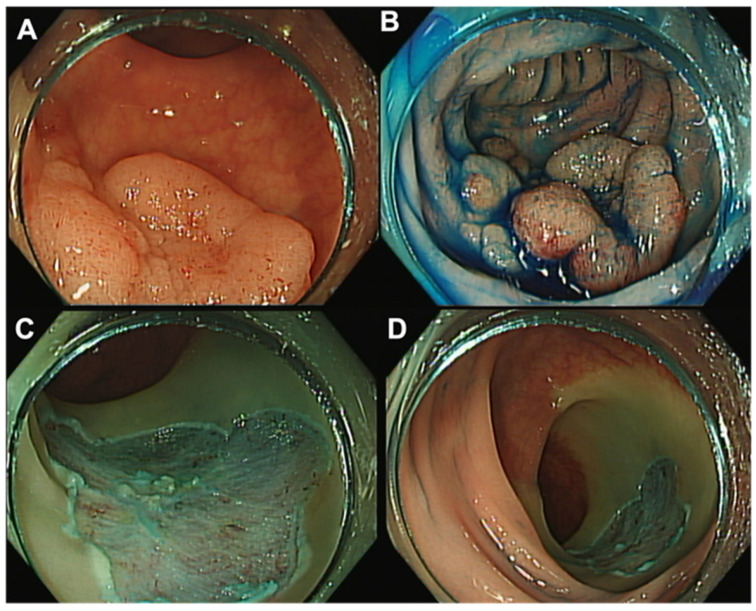
Rectal endoscopic mucosal resection (EMR). (**A**) Rectal LST-G with 30 mm. (**B**) Injection of saline–epinephrine and methylene blue for submucosal lifting. (**C**,**D**) Post-piecemeal mucosal resection with a snare.

EMR is a minimally invasive endoscopic technique that has been used successfully to remove benign and early malignant colorectal lesions in recent years [7,8,9]. EMR is performed with an endoscope and a snare, after submucosal injection (Figure 3). The submucosal injection is used to create a cushion between the abnormal tissue and the underlying muscle layer, facilitating the removal of the tissue without causing damage or bleeding. Once the cushion is created, the snare is used to capture and remove the tissue in a piecemeal or en-bloc fashion. Piecemeal resection involves removing the tissue in several pieces, while en-bloc resection involves removing the tissue in one piece.

The technique allows for the complete removal of the lesion in a minimally invasive manner, which results in minimal discomfort and a quick recovery when compared to surgery [47]. EMR comprises multiple technical variants, such as inject-and-cut (using a cold or hot snare), cap-assisted, band-assisted, underwater, gel-immersed, and hybrid techniques. Numerous studies with these variants have demonstrated that en-bloc or p-EMR can effectively and safely remove the majority of sessile or non-polypoid colorectal lesions [48,49,50,51,52].

However, while EMR is generally safe and effective, it is important to note that it may not be appropriate for all rectal lesions. As discussed, these lesions tend to be larger, which is an important limitation for this procedure [53], and the specific characteristics of the area limit its efficacy [10,54]. As indicated in several guidelines [7,9,21], lesions greater than 20 mm with signs of SMIC or non-granular lesions should be removed by ESD. It is also important to consider that EMR has a higher rate of piecemeal resection, which is linked to higher recurrence rates than en-bloc resection, which is associated with an increased risk of malignancy [7,8,9]. Overall, EMR is a safe and effective endoscopic technique for the removal of rectal lesions that are smaller than 20 mm with no signs of SMIC or a morphology suspected of malignancy. It allows for a complete resection of the lesion with minimal discomfort and a quick recovery time, with a relatively shallow learning curve and an inferior cost when compared to ESD [55]. However, in the rectum and anorectal junction, its efficacy may not be that favorable when compared to the results obtained by ESD and TAMIS, raising uncertainty about which procedure is optimal for treating lesions in this area.

### 4.2. Endoscopic Submucosal Dissection

**Figure 4:**
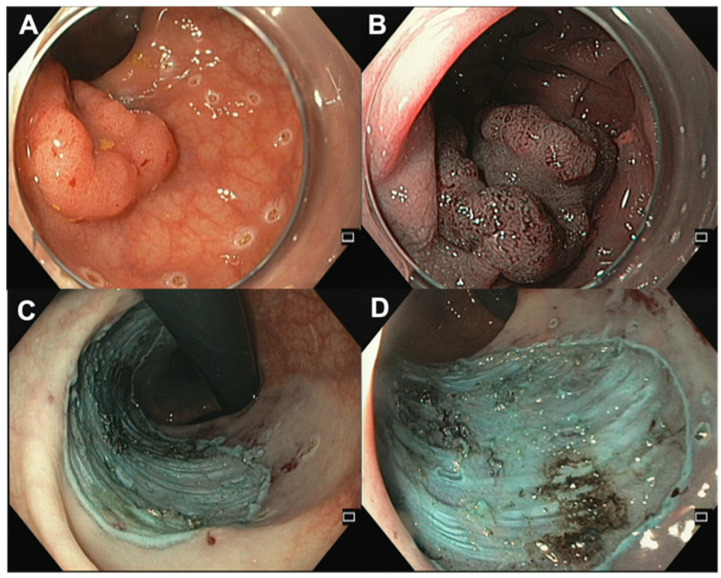
Endoscopic submucosal dissection. (**A**) Rectal LST (NICE 2, JNET 2B, Kudo Vi) with a central depression of 25 mm. (**B**) Central depression via NBI. (**C**,**D**) Lesion site after ESD in retroflexed position and frontal view.

In recent decades, the endoscopic treatment of premalignant and malignant colorectal lesions has undergone a continuous evolution, leading to the development of ESD (Figure 4). While ESD was initially created in Japan for the removal of superficial carcinomas in the upper digestive tract, Western regions have predominantly used ESD for treating colorectal lesions. This is due to several reasons, such as variations in gastrointestinal tract neoplasia among Western and Eastern populations and a lack of training opportunities in the stomach [8,9,56]. Nevertheless, the technical challenges, the need for extensive training [55] for medical and nursing personnel, and the higher complication rate compared to traditional EMR have limited its widespread adoption in Western countries [57,58]. However, in recent years, this technique has become increasingly popular due to its increased effectiveness in treating rectal and anorectal lesions, which are often difficult to remove by conventional endoscopic methods, as previously discussed. The ESD procedure is performed using endoscopes equipped with a variety of instruments, such as a high-frequency electrosurgical knife, endoscopic caps, and coagulation forceps. Briefly, a submucosal cushion is performed by a submucosal injection, followed by a mucosal incision with a dedicated knife to gain access to the submucosal layer, and finally the dissection of the submucosal fibers beneath the lesion, in order to retrieve the target lesion in a single piece.

As with any medical procedure, there is a risk of complications, such as bleeding or perforation of the digestive tract, and the duration of the procedure is usually higher than that of EMR [59]. Therefore, it is important to carefully evaluate the risks and benefits of ESD on a case-by-case basis and ensure that the procedure is performed by a qualified and experienced endoscopist. Overall, ESD is a promising technique for the removal of early-stage gastrointestinal tumors along the GI tract, being specially indicated in the esophagus and stomach, where its advantages clearly surpass the risks. As shown by Probst et al. [55], there were two study periods (2004–2013 and 2013–2016) in which, for SMIC lesions, the en-bloc resection rate was 73% and 91%, respectively. The R0 rates were 55% and 76%, and curative resection was 14% and 48%, respectively. In benign lesions, the en-bloc resection rate was 75% and 91%, with R0 rates of 55% and 85% and R1 rates of 45% and 15%. The study also reported a decrease in the rate of bleeding from 8% to 2%. The authors reported two cases of perforation (0.8%). The recurrence rate when en-bloc resection was not accomplished was 26% and 0.5% after successful en-bloc resection of the lesion. This suggests that, as the technique evolves and endoscopists gain experience, the outcomes can improve with low rates of complications.

### 4.3. Transanal Minimally Invasive Surgery (TAMIS)

Transanal minimally invasive surgery is a novel surgical technique that has been increasingly recognized in recent years as a minimally invasive approach to rectal surgery. TAMIS is a form of TEM, which was first introduced in the early 1980s [60]. The development of TAMIS is a response to the limitations of TEM, which required specialized equipment and expertise, and was associated with a steep learning curve and a requirement of considerable investment in specific equipment [54]. TAMIS is a minimally invasive surgery that allows for the removal of rectal tumors through the anus. It is primarily used for the resection of rectal polyps and early-stage rectal cancers. 

**Figure 5:**
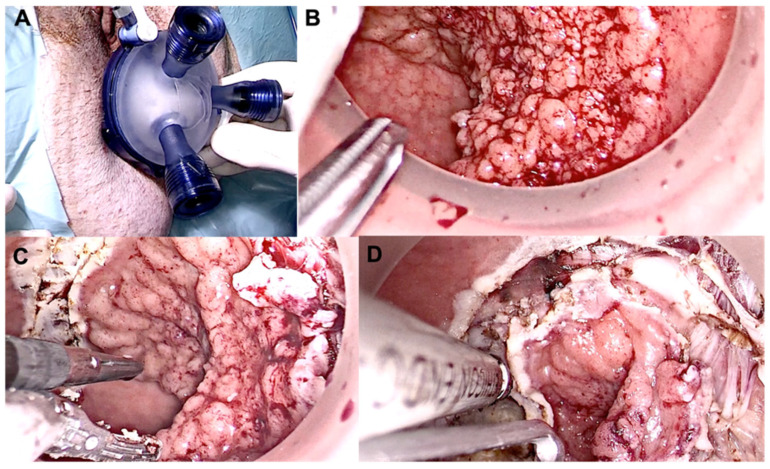
Intra-operative view of TAMIS during local excision of a distal tumor. (**A**) Glove port; (**B**) Lesion pre incision; (**C**,**D**) Dissection of the lesion.

The procedure is performed under general or regional anesthesia. A small port is inserted into the anal canal, and the rectum is inflated with carbon dioxide to obtain a clear view of the targeted lesion. Specialized laparoscopic instruments, such as a cutting device, a retractor, and scissors, are then used to remove the lesion (Figure 5). Finally, the lesion is extracted through the port. Compared to ESD, it has a relatively shallow learning curve and shorter procedure times [54]. TAMIS has several advantages over traditional open surgery including reduced pain, shorter hospital stays, and faster recovery times [60,61,62]. Compared to open surgery, the procedure is associated with fewer complications, such as bleeding and infection. Despite its benefits, TAMIS is not suitable for all patients with rectal lesions. The procedure is typically reserved for patients with early-stage rectal cancer or benign rectal polyps that are not amenable to endoscopic removal. Similar to endoscopic techniques, patients with advanced rectal cancer or those with extensive rectal disease may not be candidates for TAMIS and may require more invasive surgical procedures. There is still no clear evidence on how TAMIS compares to ESD in terms of effectiveness.

### 4.4. En-Bloc Resection—Is It Really Necessary?

En-bloc resection involves the removal of the entire tumor in only one fragment, encased by a continuous margin of healthy tissue. The objective of en-bloc resection is to achieve the complete removal of the lesion and minimize the risk of residual or recurrent disease, as well as allowing for a precise pathological evaluation [13,14,55]. As expected by the inherent technique procedure, the rate of en-bloc resection is higher in ESD procedures when compared to EMR, particularly in lesions above 15–20 mm [7,9,63]. En-bloc resection has several advantages over other resection techniques. One of the key benefits of en-bloc resection is that it reduces the risk of an incomplete removal of the lesion or an incomplete retrieval of all fragments for analysis [55]. It also allows for an accurate histological assessment of the margins of the resected tissue, helping one to determine a distinction between a benign and a malignant lesion, and to better assess deep submucosal invasion and lymphatic permeation. This is crucial in determining the extent of the disease and the need for further treatment; an error in this step can lead to an understaging and undertreatment of the patient [14,54]. Due to the limitations of preoperative staging and the safety and feasibility of en-bloc resections in the rectum, there is, currently, a shift away from piecemeal endoscopic mucosal resection (pEMR) towards en-bloc resection for large rectal lesions. Moreover, a recent analysis of cost-effectiveness indicates that opting for an en-bloc resection strategy may turn out to be more cost-effective than a piecemeal resection strategy for rectal lesions, as it could reduce the number of patients requiring additional endoscopic procedures (either therapeutic or surveillance) or further radical rectal surgery [64]. The same results were consistent with another cost-effectiveness study in which it was concluded that the removal of lesions in the rectum ESD for all LSTs larger than 20 mm is more cost-effective than pEMR or selective ESD [65].

## 5. Technique Comparison

### 5.1. Anorectal Junction

As previously stated, the anorectal junction is a specific location with complex characteristics [4,14,46], and a comparison between ESD and EMR is necessary. TAMIS, however, is not recommended in the ARJ, due to an increased rate of complications from post-procedural dysfunction of the anorectal sphincter [66,67,68]. Several retrospective studies have investigated the efficacy of both techniques for removing lesions in this location.

A Japanese study [14] analyzed the outcomes of ESD in 139 rectal neoplastic lesions, including 94 proximal rectal tumors and 45 rectal tumors extending to the dentate line (RTDLs). They reported an en-bloc resection rate of 96% and an R0 resection rate of 53% with respect to RTDLs. Regarding non-RTDLs, these resection rates were 90% and 64%, respectively. The perforation rate was 4% in RTDLs vs. 2% in non-RTDLs, while postoperative bleeding rates were 2% for RTDLs vs. 1% in non-RTDLs. Compared with rectal ESD, ESD for RTDLs had a longer procedure time (104 vs. 60 min), a higher en-bloc resection (96% vs. 90), a lower R0 resection rate (53% vs. 70%), and a higher frequency of high-grade fever (22% vs. 4%). Notably, no residual adenoma was observed during the first surveillance colonoscopy in the RTDL cases, and only one case was observed in the non-RTDL cases. The recurrence rate was 4% vs. 1%, during a median follow-up period of 18 months (Table 2).

These results were consistent with a study by Ferreira et al. [45], where there was a retrospective analysis of 252 rectal lesions, of which 24% were located in the ARJ, while the remaining 76% were located proximally. The authors did not find any statistically significant difference between ARJ and rectal lesions with regard to the en-bloc resection rate (100% vs. 96%), the R0 resection rate (76% vs. 75%), the curative resection rate (70% vs. 70) or the procedures’ median duration (120 min vs. 90 min). There was no statistically significant difference that concerns delayed bleeding (7% vs. 8%), perforation (0% vs. 5%), or the need for readmission (2% vs. 2%). However, anorectal stenosis and anorectal pain were significantly more frequent in ARJ lesions (5% vs. 0%, *p* = 0.003 and 9% vs. 1%, *p* = 0.002 respectively) (Table 3).

Another study from Probst et al. [13] analyzed 24 cases of RTDLs and 62 lesions distant from the dentate line, and their approach with ESD. RTDL and non-RTDL results showed comparable en-bloc resection rates (92% vs. 94%, *p* > 0.05), but the R0 resection rate was lower among RNDLs than among non-RTDLs (71% vs. 89%, *p* = 0.039). The recurrence rate among RTDLs was not statistically different from that among non-RTDLs (5% vs. 0%, *p* > 0.05). The median procedure time was 127 vs. 110 min (*p* > 0.05), and the risk of delayed bleeding (3% vs. 0%, *p* = 0.001) and post-interventional pain (33% vs. 15%, *p* = 0.07) were higher in the RTDL cases. The incidence of stricture did not differ.

Regarding EMR, multiple studies have been conducted to evaluate its efficacy in treating rectal and anorectal lesions. An Australian study [4] analyzed 24 lesions of ARJ resected by EMR and showed successful adenoma removal in all cases with a median resection time of 26 min. However, there was a high rate of intraprocedural bleeding and a focal adenoma recurrence (22%) in patients at the first surveillance colonoscopy. Lesions of the ARJ and proximal rectum had similar procedural success, adenoma recurrence, and admission rates.

Another study [47] analyzed the outcomes of 100 large LSTs of the anorectal junction (ARJ-LSTs) after their removal by EMR. The technical success rate was high (98%); however, en-bloc and R0 resection rates were not investigated. Submucosal invasive cancer was found in three cases. Long-term efficacy was investigated with surveillance colonoscopy at 6, 12, 36, and 60 months after the procedure, and the recurrence rates were 15%, 7%, 4%, and 0%, respectively. The median procedure time was 30 min. Clinically significant intraprocedural bleeding and pain was observed in 6% of cases (Table 4 and Table 5). 

### 5.2. Rectum

A prospective European study [55] evaluated the role of ESD in the treatment of 302 LNPCPs. They reported that 52 lesions had SMIC and associations with the Paris classification types (55% of the Type 0-Is lesions, 100% of the Type 0-Is+IIc lesions, 0% of the Type 0-IIa lesions, 15% of the Type 0-IIa+Is lesions, and 59% of the Type 0-IIa+c lesions; *p* < 0.001) and the LST classification types (71% of the non-granular lesions with mixed surfaces and 18% of the lesions with granular surfaces and nodules ≥10 mm). 

In a similar article, an American multicentric study [69] included 188 rectal lesions removed by ESD. The authors reported an en-bloc resection rate of 89% and an R0 resection rate of 86%. The curative resection rate was 80%. The study reported adverse events in 6% of cases, with delayed bleeding and perforation in 3% of patients. However, there were no reports of stricture or recurrence.

A Portuguese study [58] conducted a retrospective analysis that included 123 rectal epithelial neoplastic lesions in which the en-bloc and R0 resection rates were 97% and 82%, respectively. The curative resection rate was 78%. In terms of complications, a total of five (3.4%) perforations, all of a very small size, were reported and managed with hemoclips. Six (4.1%) cases of delayed bleedings, all manageable endoscopically, were also present.

Overall, these studies demonstrate the effectiveness and safety of ESD in the treatment of rectal and anorectal lesions, with high resection rates and low recurrence rates. The incidence of complications was low (with bleeding being the most frequently reported complication) but slightly higher in studies involving ESD than in studies focused on EMR. However, as expected, recurrence rates were significantly lower with ESD. 

Regarding EMR, an Italian group [10] analyzed the resection of 49 rectal lesions using a variation of EMR that involved a retroflexion technique. This procedure is more challenging in the lower rectum due to a narrow lumen and poor endoscopic view. The en-bloc resection rate was 94%, and the mean procedure time was 57 min. The most common complication was bleeding (during or after the procedure), which occurred in 37% of the patients. No major complications, namely perforation, were observed. During the follow-up period, recurrence was observed in nine patients.

In 2020, Shahidi et al. [47] focused on 313 rectal lesions, with an average size of 40 mm. Of these, 78.2% were considered granular, 11.7% were considered non-granular, and 9.1% were considered mixed. SMIC was found in 12.5% of cases. Technical success occurred in 97.1% of these lesions, but no additional information was given regarding en-bloc or R0 resection rates. Bleeding occurred in 12.4% with only one case of perforation. The recurrence rates were 14.7%, 7.1%, 2.4%, and 0% at SC1–SC4, respectively.

A recent article recently published evaluated the performance of a selective resection algorithm of large (>20 mm) rectal nonpedunculated rectal polyps (LNPRPs) [59]. They tested whether optical evaluation and covert SMIC risk stratification can be used to better decide the endoscopic modality of resection (EMR vs. ESD). 

A total of 480 LNPRPs were evaluated, where 290 followed the universal EMR algorithm (UEA) proposed by the guidelines [7,8] and 190 followed a rectum-specific selective resection algorithm (SRA). SMIC was identified in 56 lesions (12%). The duration of the procedure was significantly different between UEA and SRA, 29 vs. 45 min. The authors reported statistically significant differences in SMIC after EMR (SRA 1% vs. UEA 12% (*p* = 0.001) and curative oncologic resection rates (SRA 33% vs. UEA 6% (*p* = 0.01). No significant differences were found in terms of technical success or adverse events. Regarding recurrence, UEA showed rates of 17%, while with the use of SRA, the rate was 2% (*p* = <0.001) (Table 6).

Regarding the use of TAMIS for resecting early rectal tumors, we found two articles that compared this technique with ESD.

A Chinese study [70] evaluated a new surgical technique called colonoscopy-assisted transanal minimally invasive surgery via glove port (CA-TAMIS-GP) for the resection of early rectal tumors. The technique was compared to ESD. A total of 57 patients were included in the study, with 26 undergoing CA-TAMIS-GP and 31 undergoing ESD. All lesions were successfully dissected with negative margins. Postoperative recovery was uneventful for the majority of patients, except for three CA-TAMIS-GP patients who experienced minor hematochezia and seven ESD patients who experienced late-onset bleeding. The CA-TAMIS-GP group had a shorter procedure time, lower hemorrhage levels, and a lower average consumable cost compared with the ESD group (*p* < 0.05). During the follow-up period, no recurrence was observed in the CA-TAMIS-GP group, while three patients in the ESD group had local recurrence.

In a similar study with the same technique [67,71], 67 rectal tumors were prospectively evaluated. Patients were randomly divided into two groups: CA-TAMIS-GP (*n* = 32) and ESD (*n* = 35), the latter being the control group. All lesions were successfully dissected with negative margins. The CA-TAMIS-GP group had a shorter procedure time (50 min vs. 66 min, *p* < 0.001). Six patients in the surgical group experienced mild anal pain or discomfort after the procedure, while one patient submitted to ESD reported anal foreign body sensation (*p* = 0.048). The incidence of post-procedure was not statistically different between the two groups (9% in CA-TAMIS-GP group and 20% in ESD group, *p* = 0.310). No recurrence or long-term complications were reported in the CA-TAMIS-GP group, while one case of the ESD group developed local recurrence in the follow-up period.

## 6. Future Perspectives

ESD is a complex technique with a steeper learning curve but that assures adequate resection in lesions that have a significant risk of developing into malignant invasive neoplasms. However, it demands higher endoscopic skills, requires more time, and has a higher rate of adverse events when compared to EMR, a simpler and more available procedure that has been showing satisfactory results in the majority of colorectal lesions. TAMIS, compared to EMR, also requires more time, and it has a significant learning curve, yet it shows promising results in terms of efficacy and safety. New techniques, such as endoscopic intermuscular dissection (EID), a technique for resecting T1 rectal cancers with suspected deep submucosal invasion or even lesions invading muscularis propria, are emerging [72]. Unlike TAMIS, EID offers an alternative approach that preserves the rectal wall and future total mesorectal excision planes in rectal cancer resection. Further long-term follow-up and comparative studies are needed to evaluate the efficacy and outcomes of EID in comparison to classical ESD and TAMIS.

As Western endoscopists continue to gain experience and proficiency with ESD, it is anticipated that the rate of adverse events associated with this technique will decrease and become comparable to EMR. As a result, the rationale for preferring EMR is expected to diminish over time, as ESD proves to achieve better results with a similar complication rate. Nonetheless, until this point is reached, it is imperative to thoroughly evaluate both techniques by formulating prospective, randomized, and comparative trials. By evaluating both techniques through such trials, we can identify the most appropriate method for managing lesions and prioritize patient safety.

## 7. Limitations of the Study

Despite the comprehensive nature of this review, some limitations must be acknowledged. First, the study was restricted to articles published in English between 2012 and 2022 in recognized academic databases, namely MEDLINE/PubMed, EMBASE, and Cochran. The inclusion of only English language articles might have excluded relevant studies published in other languages. Another limitation of this article is the absence of a direct comparison between these techniques, which would facilitate the withdrawal of conclusions from it. In addition, it is important to acknowledge that the narrative review approach lacks the statistical analysis of data, so the findings and conclusions drawn may not be as robust or precise as those systematic reviews and meta-analyses. The absence of statistical analysis can limit the ability to determine the strength of the evidence or to quantify the effect sizes of the studies included in this review.

## 8. Conclusions

Early detection and treatment of pre-malignant lesions in the colon and rectum is crucial in reducing the morbidity and mortality associated with colorectal cancer. Less invasive procedures such as EMR, ESD, and TAMIS have shown effectiveness in treating early rectal and anorectal conditions. However, careful consideration of the characteristics of the lesion and the patient is necessary to determine the most appropriate approach. A reliable estimation method for submucosal invading carcinoma is essential to properly assess the risk of neoplastic invasion and select the most suitable technique for each lesion. Factors such as anatomic location, size, morphology, glandular, and vascular pattern should be carefully assessed, as they relate to the depth and extent of invasion and the likelihood of malignancy.

This review focuses on rectum and anorectal junction neoplasms, as these lesions have particularities that require individualized management. EMR can be effective for smaller rectal lesions with no signs of invasion, while larger lesions with signs of submucosal invasion or suspicious for malignancy may require en-bloc resection or ESD. Indeed, in recent years, ESD has been increasingly used in colorectal lesions in the West and has shown a lower rate of recurrence compared to EMR. TAMIS is also a possibility for managing rectal lesions, but more comparative studies are needed. Overall, these techniques offer effective and safe alternatives to traditional surgical interventions for the removal of rectal and anorectal lesions, but the choice of technique should be made carefully due to differences in malignancy risk and the unique anatomical and physiological characteristics of the anorectal area. Therefore, a specific approach and clinical algorithms are necessary for rectal and anorectal junction lesions. 

By individualizing the management of rectal and anorectal junction lesions, outcomes can be improved, and the morbidity and mortality associated with colorectal cancer can ultimately be reduced.

## Figures and Tables

**Table 1:** Review process screening criteria.

Stage	Criteria	Number of Articles
Initial search by combination of the key terms	Published in a peer-reviewed journal, in English, between 2012–2022, after reading the title and abstract:	
	MEDLINE/PubMed	156
	Cochrane	96
	EMBASE	31
Additional search	Selection of other relevant articles in the field	10
Full-text screening	In depth analysis of selected articles	112

**Table 2:** Comparison of the success of ESD, its recurrence rates and morbidity in lesions between RTDL and tumors in the proximal rectum. Adapted from Imai et al. [14].

	RTDL (*n* = 45)		Proximal Rectal (*n* = 94)		*p*
	Frequency	95%CI	Frequency	95%CI	
Procedure time, median (range), minutes	104 (25–420)	109–171	60 (20–326)	69.3–94.3	<0.001
Resection rates, *n* (%)					
En-bloc	43 (95.6)	85.1–98.8	85 (90.4)	82.8–94.9	0.50
R0	24 (53.3)	39.1–67.1	60 (63.8)	60.3–78.5	0.019
R1	1 (2.2)	0.4–11.6	11 (11.7)	6.7–19.8	
Recurrence rate, *n* (%)	2 (4.4)	1.3–15.4	1 (1.1)	0.2–5.8	0.26
Perforation, *n* (%)	2 (4.4)	1.2–14.8	2 (2.1)	5.9–7.4	0.59
Postoperative bleeding, *n* (%)	1 (2.2)	0.4–11.5	1 (1.1)	1.9–5.8	0.24
High grade fever (>38 °C), *n* (%)	10 (22.2)	12.5–36.3	4 (4.3)	1.7–10.4	0.002

**Table 3:** Results obtained for ESD in the rectum and anorectal junction. Adapted from Ferreira et al. [45].

	All Lesions (*n* = 252)	ARJ Lesions (*n* = 60)	Rectal Lesions (*n* = 192)	*p*-Value
Procedural duration median (range), min	90 (60–150)	120 (70–160)	90 (60–130)	0.072
Resection rates, *n* (%)				
En-bloc resection, *n* (%)	240 (97)	58 (100)	182 (96)	0.204
R0 resection, *n* (%)	187 (75)	44 (76)	143 (75)	0.531
Curative resection, *n* (%)	172 (70)	40 (70)	132 (70)	0.920
Delayed bleeding, *n* (%)	20 (8)	4 (7)	16 (8)	0.709
Perforation, *n* (%)	10 (4)	0	10 (5)	0.075
Pain, *n* (%)	7 (3)	5 (9)	2 (1)	0.002
Stenosis	3 (1)	3 (5)	0	0.003

**Table 4:** Comparison of the results obtained with the use of EMR in the rectum and anorectal junction. CSIPB: clinically significant intraprocedural bleeding; CSPEB: clinically significant postendoscopic mucosal resection bleeding. Adapted from Shahidi et al. [47].

	All Rectal LSLs (*n* = 413) *n* (%)	ARJ-LSLs (*n* = 100) *n* (%)	Rectal LSLs (*n* = 313) *n* (%)	*p*-Value
Duration (min), median (IQR)	25 (15–50)	30 (15–55)	25 (12–50)	0.045
Technical success, *n* (%)	402 (97.3)	98 (98.0)	304 (97.1)	1.000
CSIPB, *n* (%)	24 (5.8)	6 (6.0)	18 (5.8)	0.926
Deep mural injuries III–V, *n* (%)	14 (3.4)	0 (0.0)	14 (4.5)	0.027
Pain, *n* (%)	15 (4.3)	5 (6.1)	10 (3.8)	0.366
CSPEB, *n* (%)	32 (7.8)	11 (11.1)	21 (6.8)	0.162
Delayed perforation, *n* (%)	1 (0.2)	0 (0.0)	1 (0.3)	1.000

**Table 5:** Outcomes for the use of EMR with descriptions of the results obtained in follow-up colonoscopies. NA: not applicable; SC: surveillance colonoscopy. Adapted from Shahidi et al. [47].

Outcomes of Endoscopic Mucosal Resection				
	All Rectal LsLs (*n* = 413) *n* (%)	ArJ-LsLs (*n* = 100) *n* (%)	Rectal LsLs (*n* = 313) *n* (%)	*p* Value
SC1				
Eligible (*n*)	331	86	245	
Underwent SC1, *n* (%)	289 (87.3)	78 (90.7)	211 (86.1)	
Months to SC1, median (IQR)	5 (4–7)	5 (4–7)	5 (4–7)	
Recurrence at SC1, *n* (%)	43 (14.9)	12 (15.4)	31 (14.7)	0.883
Surgery at SC1, *n* (%)	1 (0.3)	0 (0.0)	1 (0.5)	
SC2				
Eligible (*n*)	264	69	195	
Underwent SC2, *n* (%)	215 (81.4)	59 (85.5)	156 (80.0)	
Months to SC2, median (IQR)	19 (14–23)	19 (14–23)	18 (15–23)	
Recurrence at SC2, *n* (%)	15 (7.0)	4 (6.8)	11 (7.1)	1.000
Surgery at SC2, *n* (%)	1 (0.5)	1 (1.7)	0 (0.0)	
SC3				
Eligible (*n*)	162	37	125	
Underwent SC3, *n* (%)	111 (68.5)	27 (73.0)	84 (67.2)	
Months to SC3, median (IQR)	40 (29–53)	39 (28–57)	41 (29–51)	
Recurrence at SC3, *n* (%)	3 (2.7)	1 (3.7)	2 (2.4)	0.570
Surgery at SC3, *n* (%)	0 (0.0)	0 (0.0)	0 (0.0)	
SC4				
Eligible (*n*)	40	10	30	
Underwent SC4, *n* (%)	31 (77.5)	10 (100.0)	21 (70.0)	
Months to SC4, median (IQR)	55 (41–69)	54 (33–83)	56 (42–69)	
Recurrence at SC4, *n* (%)	0 (0.0)	0 (0.0)	0 (0.0)	NA
Surgery at SC4, *n* (%)	0 (0.0)	0 (0.0)	0 (0.0)	

**Table 6:** Procedural Outcomes of different algorithms of treatment. Values are *n* (%), median (interquartile range), or n. SC1: surveillance colonoscopy 1; SRA: selective resection algorithm; UEA: universal endoscopic mucosal resection algorithm. LNPRPs that underwent EMR. Adapted from Shahidi et al. [59].

	Overall LNPRPs (*n* = 480)	UEA LNPRPs (*n* = 290)	SRA LNPRPs (*n* = 190)	*p* Value
Duration, min	30 (15–60)	29 (15–50)	45 (25–78)	<0.001
Technical success	468 (97.5)	280 (96.6)	188 (98.9)	0.137
Margin thermal ablation	164 (41.7)	66 (22.8)	98 (95.1)	<0.001
Deep mural injury III–V	23 (4.8)	12 (4.1)	11 (5.8)	0.407
CSPEB	40 (8.3)	21 (7.2)	19 (10.0)	0.285
Delayed perforation	1 (0.2)	1 (0.3)	0 (0.0)	1.000
SC1 Eligible	393	244	149	
Underwent SC1	360 (91.6)	233 (95.5)	127 (85.2)	<0.001
Months to SC1	6 (5–8)	5 (4–7)	7 (6–9)	<0.001
Recurrence	42 (11.7)	40 (17.2)	2 (1.6)	<0.001

## Data Availability

Not applicable.
